# Magnitude of Anemia and Its Associated Factors among Pregnant Women Attending Antenatal Care at Najo General Hospital, Northwest Ethiopia

**DOI:** 10.1155/2020/8851997

**Published:** 2020-10-19

**Authors:** Wakshuma Gari, Arega Tsegaye, Tsige Ketema

**Affiliations:** Jimma University, College of Natural Sciences, Department of Biology, Jimma, Ethiopia

## Abstract

Anemia is one of the major causes of morbidity for pregnant women in resource-limited regions. Yet robust research-based evidence on this vital public health problem in remote areas where the problem could be massive is quite limited in Ethiopia, one of the developing countries. Thus, this study is aimed to assess the magnitude of anemia and its associated risk factors among pregnant women attending one of the health facilities in Ethiopia. A facility-based cross-sectional study design was employed in 2019. A total of 384 pregnant women attending the antenatal care (ANC) unit of Najo General Hospital, Northwest Ethiopia, were included in the study. Their sociodemographic characteristics, and medical, obstetric, and gynecological history were collected using pretested interview questionnaires. Blood samples were collected from each participant for the determination of malaria parasite and hemoglobin (Hb) level. In addition, stool samples were collected for examination of intestinal parasites. Data were analyzed using Statistical Package for Social Science (SPSS) software version 25. The overall magnitude of anemia among pregnant women was 37.8% (95% CI, 32.8%–42.3%). The proportion of mild anemia, moderate anemia, and severe anemia was 24%, 11%, and 2.3%, respectively. Some variables such as absence of malaria infection (AOR: 0.195, 95% CI: 0.066–0.576), lack of history of abortion (AOR: 0.469, 95% CI: 0.265–0.830), and absence of history of anemia (AOR: 0.227, 95% CI: 0.134–0.385) were identified as protective variables of anemia during pregnancy, while urban residence (AOR: 1.753, 95% CI: 1.013–3.034) was unexpectedly found as a predisposing factor. Despite the higher number of anemic pregnant women observed in the current study, pregnancy-associated anemia is moderate public health importance in the study area.

## 1. Introduction

During pregnancy, anemia is a major public health problem in developing countries. The number of pregnant women affected by this health problem globally is estimated to 41.8%, with the highest number of cases in Africa (57.1% or 17.2 million) [1], while the prevalence is 18% in developed countries [2]. Its prevalence and severity could vary among different groups of population. According to the Ethiopian Demographic and Health Survey Report of 2016, the prevalence of anemia in pregnant women was 24% [3]. Other studies recently reported from different places of Ethiopia showed that the prevalence of anemia among pregnant women could range from 9.7% to 56.8% in central and Eastern part of the country, respectively [4, 5]. Untreated anemia in the pregnant women has severe consequences on health, social, and economic development of the nation in general [1]. Some of the associated threats are increased such as maternal morbidity and mortality (mainly it happens when the anemia is severe), low birth weight, preterm delivery, fetal anorexia, intrauterine growth restriction, perinatal mortality, and maternal mortality [6–9]. This health problems, unless diagnosed early and treated, could have multifactorial risks to the pregnant women.

The major cause of anemia in pregnant women is shortage of red blood cells (RBCs). This happens when there are deficiencies of Iron, folate, vitamin B12, and vitamin A, intestinal or blood parasite infections, and other chronic illness [6, 9–11]. Despite presence of many reports on high prevalence of anemia among pregnant women in Ethiopia, lack of regular surveillance and management plan at all levels are among major concerns for pregnant women in general and those living in remote areas of the country in particular. Therefore, this study was designed to give an insight on the current prevalence of anemia and its determinants in one of the remote areas never surveyed for anemia in pregnant women in Ethiopia.

## 2. Materials and Methods

### 2.1. Description of the Study Area

The study was conducted at Najo General Hospital, Northwest Ethiopia, in May, 2019. The study area is located in the Northwest of Ethiopia at a distance of 515 km from Addis Ababa, the capital city of Ethiopia. Geographically, the study site is located at latitude and longitude of 9°30ꞌ 0ꞌꞌ N and 35°30ꞌ 0ꞌꞌE, respectively. The hospital has been serving population estimated to about 1,000,000 (223,000 of whom were females in reproductive age) (unpublished data, annual report of Nejo General Hospital, 2017/18).

### 2.2. Operational Definitions

   Light/normal menstrual level: menstrual cycle in 21–35 days of duration, with bleeding lasting an average of 5 days and total blood flow between 25 and 80 mL.
Heavy menstrual level: a menstrual bleeding with a total menstrual flow of >80 ml per cycle, soaking a pad/tampon at least every 2 hours, or bleeding lasting for >7 days [12].

### 2.3. Study Participants and Design

A facility-based cross-sectional study design was employed. All pregnant women who live in the catchment area of the hospital and attending an ANC during the study period were included in the study. Pregnant women who were seriously ill, with known multiple pregnancies, and had history of chronic disease such as tuberculosis (TB) and human immunodeficiency virus (HIV)/acquired immune deficiency syndrome (AIDS) were excluded from the study.

### 2.4. Sample Size and Data Collection Method

The sample size was calculated considering 95% CI, and using 56.8% prevalence rate reported from Eastern Ethiopia [4], the highest proportion of anemia, and ±5% precision [13]. Considering 10% nonresponse rate, a total of 414 samples were computed, but only 384 pregnant women attended the ANC during the study period and fulfilled the inclusion criteria were recruited using simple random sampling technique. Then, data were collected using the pretested interview questionnaire comprising questions related to sociodemographic characteristics, obstetric and gynecological history, and clinical feature of the participants [14].

### 2.5. Sample Collection and Examination

The participants were given a universal bottle and instructed on how to do proper stool collection. About 1gm of stool sample was collected from each study participant. Some portion (∼50 mg) of the sample was used for direct wet mount preparation and then examined under a microscope within 10 minutes of the collection. The remaining portion of the fecal sample was preserved in formalin and then after used for formal-ether sedimentation concentration technique. In addition, on the same date, few drops of blood samples were collected from pricked finger of the participants on a glass slide for malaria diagnosis. Accordingly, a thin smear was stained using Giemsa (10%) right after fixed in methanol. Then, the slides were examined under a microscope (high magnification lens). Again, small amount of blood (∼100 *µ*l) was drawn into a microcuvette to measure hemoglobin level using portable HemoCue Hb301 (HemoCue, Haemoglobinometrer, Angelholm, Sweden). Moreover, for each pregnant woman, the body mass index (BMI) was calculated using their height and weight data.

### 2.6. Data Analysis

Data were checked for their completeness, then entered into Microsoft Excel spreadsheets. Then, data were exported to software (Statistical Package for Social Science (SPSS)) version 25.0 for analysis. Descriptive statistics such as percentage and standard deviation (SD) were used to compute some basic sociodemographic characteristics, and obstetric and gynecologic, and clinical data. Bivariate and multivariate logistic regression analyses were conducted to identify independent predictors of anemia. Bivariate analysis was conducted to select candidate variables (*P* < 0.25) for multivariate analysis, and the later was used to identify independent predictors of anemia. The goodness of fit of the final logistic model was tested using the Hosmer and Lemeshow test at *P* > 0.05. 95% confidence interval (CI) was used for all analysis, and the significant level was considered at *P* < 0.05.

## 3. Results

### 3.1. Description of Study Participants and Response Rate

More than half of the pregnant women, 220 (57.3%), who participated in this study were found in the age range between 15–24 years, with a mean age of 23.57 years (SD ± 3.8 year). Majority of them were married, 378 (98%), from the Oromo ethnic group, 379 (98.7%), had ≤2 family size, 195 (51%), followers of protestant religion, 228 (59.6%), attended at least primary school, 151 (39.3%), housewives, 253 (66%), and urban dwellers, 276 (72%) (Table 1).

### 3.2. Obstetric and Medical Factors

Finding from the blood film and stool sample examination revealed that, a total of 20 (5.2%) pregnant women were infected with malaria and 4 (1%) were infected with different intestinal parasites. Most of the pregnant women involved in the study responded that they have history of light menstrual level, 293 (76.3%), and history of using contraceptive, 329 (85.7%), and some had history of abortion, 113 (29.4%). Regarding the obstetrical history, 144 (37.5%) pregnant women were multigravidas, 122 (44.8%) were nullipara, and 170 (44.3%) had ≤ 1 year birth interval. The gestational age for 116 (30.2%), 95 (24.7%), 102 (26.6%), and 71 (18.5%) was at first, second, third, and fourth trimester of pregnancy, respectively. A total of 336 (87.5%) participants had normal BMI (Table 2).

### 3.3. Magnitude of Anemia

Concerning the anemic status of the pregnant women, 145 (37.8%, 95%CI: 32.8%–42.3%) were anemic (their Hb level was <11 g/dL). About 93 (24.2%) of them were with mild anemia (Hb level: 10–11 g/dL), 43 (11.2%) had moderate anemia (Hb level: 7–9.9 g/dL), and the rest, 9 (2.3%), were diagnosed with severe anemia (Hb level: ≤7 g/dL).

### 3.4. Factors Associated with Anemia

Findings of bivariate and multivariate regression analyses showed that almost all sociodemographic characteristics including age, marital status, family size, monthly income, and occupation did not show significant (*P* > 0.05) association with the incidence of anemia, except for residence of the participants, where prevalence of anemia was significantly higher among pregnant women's residing in urban area than in rural (Table 3).

Medical factors such as lack of malaria infection, absence of history of abortion, and history of anemia showed significant association (*P* < 0.05) with the current low prevalence of anemia among the pregnant women. The rest such as helminthic infection, history of contraceptive use, intensity of menstrual level, trimester, body mass index (BMI), birth space, parity, gravidity, and type of contraceptives being used did not show significant (*P* > 0.05) association with occurrence of anemia among the pregnant women (Table 4). In this last step of multiple logistic regression analysis, four variables were found to be statistically independently associated with anemia in pregnancy. Accordingly, absence of malaria infection (AOR = 0.195, 95% CI: 0.07–0.58, *P*=0.003), absence of history of abortion (AOR = 0.47, 95% CI: 0.26–0.83, *P*=0.009), and absence of anemia history (AOR = 0.23, 95% CI: 0.134–0.385, *P* < 0.001) were identified as protective variables from anemia, while urban residence (AOR: 1.75, 95%CI: 1.01–3.034, *P*=0.045) was found as exposing variable to anemia during pregnancy. Pregnant women with a history of anemia, abortion, and infected with malaria could be potentially vulnerable to anemic condition. Also, pregnant women who resided in urban had 1.75 times more likely to be anemic compared to those who live in rural residence (Table 4).

## 4. Discussion

Magnitude of anemia documented in this study was 37.8% (95% CI: 32.8%–42.3%), from which 2.3% were due to severe anemia. Conferring to the Ethiopia Demographic and Health Survey (EDHS) report, about 24% of Ethiopian pregnant women aged 15–49 years were anemic, among which 18%, 5%, and 1% were mildly anemic, moderately anemia, and severely anemic, respectively [3]. In line with the 2016 EDHS survey, the proportion of anemic pregnant women of reproductive age documented in the current study area was much higher than the national survey. Also, the severe anemia cases observed were higher than the reported national survey and also reports from other African countries such as Nigeria (0.3%), Kenya (0.8%), Libya (1%), and Tanzania (2.1%) [11, 15–17]. However, it showed consistent pattern with reports from Ethiopia (Southern (39.94%), Central (36.6%), and Northwestern Ethiopia (36%)) [15, 18, 19]. The discrepancy might be due to difference in socioeconomic status, living style, prevalence of parasitic infection (e.g., malaria), geographical, study time difference (currently improved health facilities are accessible to the pregnant women with better awareness), and interventional activities (such as iron tablet supplementation) being undertaken in different countries [20].

Various sociodemographic factors were found as determinants of anemia in pregnant women [11,15]. Some of these factors are residence, educational and economic status, age of the mother, family size, and occupational status of the mother [20, 21]. In this study, some of these variables did not show significant association with the occurrence of anemia, except for urban residents, which was strongly associated with anemia among pregnant women. This finding agreed with the finding reported from a study conducted at the Nekemte health center, Ethiopia [19], where sociodemographic variables did not show statistically significant association with anemia. Absence of malaria infection was found as protective variable to anemia. It is strongly accepted that malaria is one of the major causes of anemia in tropics [22]. Hemolysis that occurs due to rupturing of malaria infected and noninfected red blood cells is responsible for the development of anemia [22, 23]. Additionally, during pregnancy, malaria tends to be more a typical in presentation. This could be due to the hormonal, immunological, and hematological changes during pregnancy [24]. Hormonal changes during pregnancy reduced synthesis of immunoglobulins (Igs) and reduced reticuloendothelial function [25, 26]. These changes result in loss of acquired immunity to malaria, making the pregnant women more vulnerable to malaria infection as well as the parasitaemia tends to be 10 times higher and, as a result, malaria tends to be more severe in pregnancy compared to the nonpregnant population [25, 27].

In resource-limited countries such as Ethiopia, the cause of anemia during pregnancy is multifactorial. The degree of bleeding resulted from complicated unsafe abortion [28–30] could be manifested by incidence of anemia [31, 32]. The risk will increase to a greater extent if the woman was anemic before she got pregnant [29]. In support of this view, findings of this study showed that pregnant women without a previous history of anemia and lack history of abortion are less likely to become anemic. In Africa, 60% of unsafe abortions generally occur in women aged below 25 years and 40% occur in the adolescent age [31]. Likewise, most of the pregnant women involved in the current study were found aged between 15 and 24 years, and the number of pregnant women with the experience of abortion was significantly higher in this age group. Unlike other reports from the same country and abroad [4], obstetric and gynecological factors such as parity, trimesters, history of menstrual level, history of contraceptive use, and gravidity did not show significant association with anemia in the current study. The observed difference might be due to the fact that high percentage of pregnant women participated in the study was nullipara (44.8%) compared to other parities. However, it was similar with reports from urban area of Eastern Ethiopia, Saudi Arabia, and India [4, 33, 34], where most of the obstetric and medical factors have no association with incidence of anemia.

## 5. Conclusion

The overall prevalence of anemia among pregnant women (37.8%) in the current study was high. According to the WHO classification of anemia, this prevalence could be considered as a moderate public health problem. However, it is higher than the national anemia prevalence (24%) reported by the Ethiopian Demographic Health Survey (EDHS) in 2016. Urban residence, presence of malaria infections, having history of abortion, and history of anemia were identified as predisposing factors of anemia among the pregnant women, whereas absence of these variables was found protective against this public health problem.

## 6. Ethical Consideration

The study was ethically approved by Research and Ethical Review Board of Jimma University, College of Natural Sciences. Oral consent agreements and written informed consent were obtained from all study participants prior to data collection; anemic (mild and moderate) pregnant women were consulted to eat iron-rich foods and given iron tablets. Also, those with severe anemic condition got treated at the hospital.

## Figures and Tables

**Table 1:** Sociodemographic characteristics of pregnant women attending ANC of Najo General Hospital, 2019.

Variable	Category	Frequency (%)
Age	15–24	220 (57.3)
	25–30	135 (35.2)
	>30	29 (7.6)

Marital status	Married	376 (98)
	Others^a^	8 (2.1)

Ethnicity	Oromo	379 (98.7)
	Others^**b**^	5 (1.3)

Family size	≤2	195 (51)
	2–4	178 (46.4)
	>4	11 (2.9)

Religion	Orthodox	133 (34.6)
	Muslim	21 (5.5)
	Protestant	228 (59.4)
	Wakefeta	2 (0.5)

Educational status	Illiteracy	25 (6.5)
	Primary school (grade 1–8)	151 (39.3)
	Secondary school (grade 9–12)	144 (37.5)
	Higher education (above grade 12)	64 (16.7)

Occupation	Employed	62 (16.1)
	Housewife	253 (66)
	Farmer	29 (7.6)
	Others^c^	40 (10.4)

Residence	Urban	276 (72)
	Rural	108 (28.1)

^a^Others = single and widowed; ^b^Others = Amhara and Gurage; ^c^Others = self-employee, daily laborer, and business women.

**Table 2:** Obstetric and medical factors of pregnant women attending ANC of Najo General Hospital, 2019.

Variable	Category	Frequency (%)
Helminthic infected	Yes	4 (1)
	No	380 (99)

Malaria infected	Yes	20 (5.2)
	No	364 (94.8)

History of use contraceptive	Yes	329 (85.7)
	No	55 (14.3)

History of menstrual level	Heavy	91 (23.7)
	Light	293 (76.3)

Trimesters	First	116 (30.2)
	Second	95 (24.7)
	Third	102 (26.6)
	Fourth	71 (18.5)

History of abortion	Yes	113 (29.4)
	No	271 (70.6)

Body Mass index	Overweight (>25 Kg/m^2^)	39 (10.2)
	Normal (18.5–25 Kg/m^2^)	336 (87.5)
	Underweight (18.5 Kg/m^2^)	9 (2.3)

Birth spacing	≤1 year	170 (44.3)
	2–3 year	65 (16.9)
	>3 year	149 (38.8)

Parity (number of deliveries)	Nullipara	172 (44.8)
	Primipara	113 (29.4)
	Multipara	97 (25.3)
	Grand multipara	2 (5)

Hemoglobin level	≤7 g/dL	9 (2.3)
	7–9.9 g/dL	43 (11.2)
	10–11 g/dL	93 (24.2)
	>11 g/dL	239 (62.2)

Gravid	First	144 (37.5)
	Second	117 (30.5)
	Third	76 (19.8)
	Fourth	35 (.9.1)
	≥fifth	12 (3.1)

History of contraceptive use	Depo-Provera	182 (47.4)
	Implanon	89 (23.2)
	Loop	11 (2.9)
	Combined contraceptive	9 (2.3)
	Depo and Implanon	35 (9.1)

**Table 3:** Association of sociodemographic factors and anemia among pregnant women attending ANC of Najo General Hospital, 2019.

Variables	Category	Anemic	Not anemic	COR (95% CI)	*P* value	AOR (95% CI)	*P* value
Age	15–24	91	129	0.685 (0.438–1.074)	0.099	0.765 (0.45–1.3)	0.325
	25–30	44	91	1	1	1	1
	>30	10	19	0.919 (0.394–2.14)	0.844	1.76 (0.6–5.2)	0.309

Marital status	Married	140	236	2.81 (0.661–11.94)	0.162	2.5 (0.41–15.34)	0.323
	Others	5	3	1		1	1

Family size	≤2	68	127	3.27 (0.924–11.56)	0.066	2.14 (0.42–10.97)	0.361
	2–4	70	108	2.7 (0.762–9.56)	0.124	1.45 (0.3–7.1)	0.649
	5–6	7	4	1		1	1

Educational status	Illiteracy	12	13	0.74 (0.29–1.88)	0.528		
	Primary school	55	96	1.19 (0.656–2.17)	0.561		
	Secondary school	52	92	1.21 (0.66–2.21)	0.535		
	> grade 12	26	38	1			

Occupation	Employed	23	39	1		1	1
	Housewife	102	151	0.73 (0.31–1.7)	0.462	1.13 (0.59–2.17)	0.712
	Farmer	8	21	0.63 (0.31–1.3)	0.216	2.73 (0.82–9.08)	0.101
	Others	12	28	1.12 (0.39–3.24)	0.827	2.36 (0.82–6.78)	0.111

Residence	Urban	95	181	1.64 (1.045–2.58)	0.032^*∗*^	1.75 (1.01–3.03)	0.045^*∗*^
	Rural	50	58	1		1	1

NB: ^*∗*^indicates significant difference.

**Table 4:** Association of medical factors and anemic conditions among pregnant women attending ANC of Najo General Hospital, 2019.

Variables	Category	Anemic	Not anemic	COR (95% CI)	*P* value	AOR (95% CI)	*P* value
Helminthic infection	Yes	3	1	1	1	1	1
	No	142	238	0.2 (0.02–1.9)	0.164	0.095 (0.01–1.02)	0.052

Malaria infection	Yes	12	8	1	1	1	1
	No	133	231	0.38 (0.15–0.96)	0.041^*∗*^	0.195 (0.07–0.58)	0.003^*∗*^

History of contraceptive	Yes	123	206	1.11 (0.62–2.0)	0.711		
	No	22	33	1	1		

Menstrual level	Heavy	36	55	0.9 (0.56–1.47)	0.685		
	Light	109	184	1	1		

Trimester	1^st^ trimester	49	67	1	1		
	2^nd^ trimester	35	60	0.89 (0.49–1.62)	0.705		
	3^rd^ trimester	33	69	1.12 (0.59–2.1)	0.733		
	4^th^ trimester	28	43	1.362 (0.72–2.56)	0.338		

History of abortion	Yes	53	60	1	1	1	1
	No	92	179	0.58 (0.37–091)	0.018^*∗*^	0.469 (0.27–0.83)	0.009^*∗*^

Birth space	≤1 year	64	106	0.91 (0.58–1.44)	0.701		
	2–3 year	28	37	0.73 (0.40–1.32)	0.299		
	>3 year	53	96	1	1		

Parity status	Nullipara	65	107	1	1		
	Primipara	41	72	1.65 (0.1–26.7)	0.726		
	Multipara	38	59	01.76 (0.11–28.8)	0.693		
	Grand multipara	1	1	1.55 (0.09–25.57)	0.758		

History of anemia	Present	62	37	0.24 (0.15–0.39)	0.000^*∗*^	0.23 (0.13–0.385)	*P* < 0.001^*∗*^
	Absent	83	202	1	1	1	1

Gravid	First	52	92	1	1	1	1
	Second	38	79	3.54 (1.02–12.32)	0.047^*∗*^	1.6 (0.8–3.2)	0.181
	Third	28	48	4.16 (1.18–14.67)	0.027^*∗*^	1.62 (0.66–4)	0.292
	Fourth	19	16	3.43 (0.95–12.45)	0.061	0.54 (0.19–1.55)	0.250
	Fifth	8	4	1.68 (0.43–6.64)	0.457	0.23 (0.04–1.26)	0.091

History of contraceptive use	DP	70	112	0.84 (0.39–1.8)	0.641		
	Implanon	36	53	0.77 (0.34–1.74)	0.526		
	Loop	3	8	1.39 (0.31–6.23)	0.666		
	Combined	1	8	6.23 (0.47–37.4)	0.311		
	DPI	12	23	1	1		

## Data Availability

The data used to support the findings of this study are all included/available in the manuscript.
